# Unmet supportive care needs and its relation to quality of life among adult acute leukaemia patients in China: a cross-sectional study

**DOI:** 10.1186/s12955-020-01454-5

**Published:** 2020-06-23

**Authors:** Yan Jie, Ying Wang, Jingyi Chen, Chunfeng Wang, Yingchun Lin, Rong Hu, Yong Wu

**Affiliations:** 1 School of Nursing, Fujian Medical University, NO.1 Xueyuan Road, Shangjie Town, Minhou County, Fuzhou City, 350122 Fujian Province China; 2 Department of Hematology, the First Affiliated Hospital of Fujian University, No. 20 of Chazhong Road, Taijiang District, Fuzhou City, 350004 Fujian Province China; 3 Department of Hematology, Fujian Medical University Union Hospital, No. 29 Xinquan Road, Gulou District, Fuzhou City, 350001 Fujian Province China

**Keywords:** Acute leukaemia, Supportive care, Cancer patients, Life quality, Needs domains, Chemotherapy

## Abstract

**Background:**

Patients with acute leukaemia (AL) usually require prolonged periods of hospitalisation. The treatment and clinical symptoms may lead to patients’ supportive care needs (SCNs) not being met and impairs their quality of life (QoL). Studies on QoL and SCNs among AL patients are limited. This study aimed to identify the unmet SCNs and its relation to QoL of adult AL patients in China.

**Methods:**

This multicentre cross-sectional study recruited 346 participants to complete a self-developed questionnaire, detailing demographic information and disease-related variables. A 34-item Supportive Care Needs Survey (SCNS-SF34) was used to identify unmet SCNs, and the Functional Assessment of Cancer Therapy-Leukaemia (FACT-Leu) questionnaire measured patients’ QoL.

**Results:**

Unmet SCN rates for the 34 items ranged from17.6 to 81.7%. Patients’ needs were high for health systems and information, but low in the sexual domain. The results reveal nine factors associated with the unmet SCNs of adult AL patients, including marital status, original residence, age, education, occupation, other diseases, chemotherapy course, disease course, and treatment stage (*p* < 0.05). The total score of the FACT-Leu negatively correlated with the SCNS-SF34 in the physical/daily living (r = − 0.527, *p* < 0.01), psychological (r = − 0.688, *p* < 0.01), sexual (r = − 0.170, *p* < 0.01), patient care and support (r = − 0.352, *p* < 0.01), and health systems and information (r = − 0.220, *p* < 0.01) domains.

**Conclusions:**

Adult AL patients exhibit a high demand for unmet SCNs, especially in the domain of health systems and information. There was a significant association between patients’ unmet SCNs and QoL. Future research should develop tailored interventions to address the unmet SCNs of adult AL patients, to further improve their QoL.

## Background

Leukaemia is a heterogeneous group of haemopoietic cancers and the tenth leading cause of cancer death worldwide [1]. According to Global Cancer Statistics 2018, there were 437,033 new cases of leukaemia in 2018, of which 309,006 (70.7%) died, corresponding to a very high mortality rate [2]. In China, there were nearly 75,300 new diagnoses of leukaemia, and approximately 53,400 leukaemia patients died, in 2015 [3]. The incidence of acute leukaemia (AL) has moderate increase from 1992 to 2017, with an estimated 26,090 new cases (including 6150 acute lymphoblastic leukemia (ALL) and 19,940 acute myeloblastic leukemia (AML)) diagnosed in the United States in 2020 [4]. The diagnosis and treatment of the disease can cause clinical symptoms and psychologically burden AL patients.

AL develops rapidly and can cause several clinical symptoms, including fever, fatigue, anaemia, bleeding, and infection [5]. Chemotherapy, as an effective treatment, can improve the overall survival rate of patients with AL [6]. However, the side effects of chemotherapy leads to patients with significant physical and psychological challenges, which affects their quality of life (QoL) [7]. Additionally, AL patients undergoing induction chemotherapy usually require prolonged periods of hospitalisation, typically lasting from three to four weeks [8, 9]. Treatment and clinical symptoms of cancer can lead to enduring negative consequences for patients, including physical, psychological, social support and financial problems [10, 11]. These negative consequences may further lead to patients’ supportive care needs (SCNs) not being met.

Due to the lack of knowledge of AL, the skills required to address symptoms can trigger multi-domain needs (information, emotional, spiritual, social, or physical needs) throughout the treatment process. SCN is broadly defined as “the provision of the necessary services for those living with or affected by cancer to meet their informational, emotional, spiritual, social, or physical needs during their diagnostic, treatment, or follow-up phases encompassing issues of health promotion and prevention, survivorship, palliation, and bereavement.” [12] Currently, most studies on SCNs for cancer patients are conducted in western countries [13], or in areas with well-funded health care systems areas, such as Hong Kong [14], Taiwan [15] and Japan [16]. SCNs may vary depending on the patient’s cultural background [13]. Patients have been reported to be better able to cope with cancer by receiving quality supportive care [17].

Today, more attention is being paid to meeting the needs of patients suffering from other types of cancer, such as prostate and breast cancer, in order to improve cancer patients’ QoL [18, 19]. Previous studies have shown that patients with AL have a low QoL [20]. Reduced QoL may be associated with psychological disorders or recurrence [21–23]. The relationship between the QoL and the SCNs of patients with cancer has recently been recognized [24]. Addressing the unmet SCNs of cancer patients in time, can improve the poor QoL of patients and increase survival rates [18, 25, 26]. Identifying unmet supportive needs can improve the psychological well-being and QoL of cancer patients, and guide clinical practice [27]. Improving the quality of care is evidence-based and addresses patients’ unmet SCNs [28]. Therefore, assessing a patient’s needs should take into account their preferences, to identify areas of unmet needs and cross-cultural influences [29].

This study aimed to: (i) investigate the unmet SCNs of adult AL patients; (ii) explore the factors associated with unmet SCNs of adult AL patients; and (iii) assess the relationship between unmet SCNs and the QoL of adult AL patients, on mainland China.

## Methods

### Study design and participants

This is a multicentre cross-sectional study, using a convenience sampling method to collect data. All AL adult patients who were referred to the departments of haematology of the three largest tertiary hospitals in Fuzhou, China, including the Fujian Medical University Union Hospital, the First Affiliated Hospital of Fujian University, and the Fujian Provincial Hospital between April 2017 and January 2018 were eligible for this study.

The selection criteria for adult AL patients included: (1) over 18 years of age; (2) a definitive AL diagnosis, based on a blood disease diagnosis and efficacy standard, (3) able to communicate fluently in Mandarin Chinese, and (4) the patient is aware of their cancer diagnosis and is willing to participate in the study. Patients were excluded if they had severe vital organ disease, or mental or cognitive disorders, such as dementia, loss of consciousness, or delirium. The proposed sample size was within five to ten times the number of items on the main scale of this study [30]. Therefore, as the Supportive Care Needs Survey form (SCNS-SF34) contains 34 items, it was estimated that 170 to 340 participants were required.

In total, 346 patients who were newly diagnosed or under therapy were invited to participate in the survey, and 325 (93.9%) completed the questionnaire. Patients who declined to participant provided reasons, including their illness (*n* = 15), lack of interest (*n* = 3), or lack of time (n = 3). Nineteen returned questionnaires were excluded from the final analyses, due to missing data. This resulted in a final sample size of 306 (88.4% of invited participants).

### Procedures

Data were collected by four dedicated investigators on each inpatient’s bed through face-to-face interviews in haematology wards. After evaluating patients for eligibility, and inviting them to participate, consultations were held with interested recruits. Potential participants were identified from the hospital’s electronic medical records. Prior to registration, all participants were informed of the objectives of the study and their rights. All participants voluntarily signed informed consent forms and were encouraged to complete socio-demographic information questionnaire, the SCNS-SF34 and FACT-Leu independently. Each questionnaire required between 10 and 30 min to complete. However, assistance from the investigator was available if participant has low vision or other disabilities. After each participant submitted the questionnaire, the investigator reviewed it and asked the participant to fill in any missing items. Imputing the missing values will be conducted when a subject completes only part of the questionnaire. In the case where less than half of the items within a domain are missing, we will impute a value that is equal to the mean for the individual of the other items in that domain [31]. The research followed the supportive care need framework [32], to explore unmet supportive care needs, which are conceptualized as influencing factors on long-term health outcomes, such as QoL.

### Measurements

A self-reported questionnaire that includes two sections, was administered to collect data. The first section obtained the sociodemographic and clinical characteristics of adult AL patients. The second section includes the Supportive Care Needs Survey form (SCNS-SF34) and the Functional Assessment of Cancer Therapy-Leukaemia (FACT-Leu) questionnaire. Participants completed the questionnaire, based on their actual experiences from the previous week.

#### Participants’ sociodemographic and clinical characteristics

A questionnaire was used to collect sociodemographic information, including age, gender, marital status, religion, education and occupation. The clinical characteristics obtained, include the type of AL, the presence of absence of other diseases, the disease course, the chemotherapy course, the treatment stage, and whether the patient received initial treatment. Patients’ medical records were collected from the patients’ electronic records, with hospital permission.

#### The Chinese version of supportive care needs survey-short form 34 (SCNs-SF34)

This study employed the SCNS-SF34 to measure the unmet SCNs of adult AL patients. The SCNS-SF34 comprises 34 items with five domains: physical/daily living (5 items), psychological (10 items), patient care and support (5 items), sexuality (3 items), and health system information (11 items). Standardised scales were used to analyse SCN domains. For each item, patients were asked to indicate their level of unmet need over the last month. Patients could rate each item on a five-point Likert scale: 1 = not applicable, 2 = satisfied, 3 = low need, 4 = moderate need, and 5 = high need. The percentage of unmet SCNs were evaluated. According to SCNS-SF34 scoring instructions [33], the standard score of each domain were calculated by n × 100/(m × (k-1)), which rescaling to a 0–100 range (m equals the number of each domain and k means the value of the maximum response for each item, n equals the result of summing the individual items, subtracting m) [34]. Higher scores indicate more unmet needs of help. Cronbach’s alpha of five domains of SCNS-SF34 ranged from 0.78 to 0.92 [18]. In this study, we divided the unmet SCNs of each domain into two groups: “no/low demand (including not applicable/satisfied/low need)” and moderate/high demand”. Across five domains, the rank of each entry and percentage of moderate/high demand of unmet SCNs were evaluated. The SCNS-SF34 has been validated in Chinese cancer patients [35].

#### The Chinese version of the functional assessment of cancer therapy-Leukaemia (FACT-Leu)

Patient QoL was measured using the Chinese version of the Functional Assessment of Cancer Therapy-Leukaemia (FACT-Leu) questionnaire [36], which contains 27 Functional Assessment of Cancer Therapy-General (FACT-G) items and 17leukemia-specific items, comprising the physical well-being (PWB, 7 items), social/family well-being (SWB, 7 items), emotional well-being (EWB, 6 items), functional well-being (FWB, 7 items) and Leukaemia-specific subscale (LEUS, 17 items). The items were assessed according to a five-point Likert scale (0 = not at all; 1 = a little bit; 2 = somewhat; 3 = quite a bit; and 4 = very much). The item scores were summed up to give the scores for each domain and the total score. Higher scores indicated better QOL. The score ranges from 0 to 176, with higher scores indicating better QoL. The internal consistency of all FACT-Leu subscales and aggregated scores was high (Cronbach’s alpha from 0.75 to 0.96). The Chinese version of FACT-Leu was shown to be reliable for measuring QoL in leukaemia patients [36].

### Statistical analysis

Data from 306 adult AL patients met the eligibility criteria and were included in this study. A database was created using EpiData3.1 software (The EpiData Association, Odense, Denmark), and the data were each input by two researchers. The analysis was performed using IBM SPSS 22.0 (Armonk, NY, USA). Descriptive statistics were used to summarize the sociodemographic and clinical characteristics of adult AL patients. Categorical variables are presented as frequencies (n) and percentages (%); continuous variables are presented as mean ± standard deviation (SD) or median (M); the interquartile range (IQR) depends on the results of the normality test. Independent-sample t-tests (for two groups) or one-way analyses of variance (for multiple groups) were conducted to analyse the influence of patients’ sociodemographic and clinical characteristics on unmet SCNs. Multivariate logistic regression was used to analyse the relevant factors affecting patients’ unmet SCNs, and the odds ratios (ORs) and 95% confidence interval (CI) were calculated. The correlation between unmet SCNs and the QoL of adult acute leukaemia patients was examined using Spearman’s rank correlation or Pearson correlation. All significance tests were two-sided and the level of significance was set at *p* < 0.05.

## Results

### Participants’ characteristics

Table 1 showed the characteristics of the adult AL patients. Of the research sample of 306 participants, the elderly AL patients (ages older than 60) accounted for 13.4%. The majority of patients (52.0%) were male and 227 (74.2%) were married. Most patients were unemployed (89.8%) and were unaffiliated with a religion (51.6%). The majority of patients (52.3%) lived in city while the rest lived in country. The vast majority (75.2%) of the patients had a Junior college education level or lower. Participants whose disease course was longer than 6 months made up 40.8% of the total. The majority (55.9%) of patients’ chemotherapy course was equal or more than 3 times. Additionally, 194 (63.4%) of the participants were diagnosed with AML, and 112 (36.6%) had ALL. The majority (55.2%) of the patients didn’t have other diseases (i.e. hypertension or diabetes). The vast majority (76.8%) of the patients were undergoing initial treatment for the condition while, all together, majority of the subjects were in the induction (46.1%) or consolidation (37.2%) treatment stage.

**Table 1:** Social-demographic information and clinical characteristics (*n* = 306)

Variables	Sample n (%)
Gender	
Male	159 (52.0)
Female	147 (48.0)
Age (years)	
14 ~ 35	137 (44.8)
36 ~ 60	128 (41.8)
> 60	41 (13.4)
Marital status	
Married	227 (74.2)
Not married^a^	79 (25.8)
Original residence	
City	160 (52.3)
Country	146 (47.7)
Religion	
No	158 (51.6)
Yes^b^	148 (48.4)
Occupation	
Farmer	81 (26.5)
General worker	35 (11.4)
Student	39 (12.7)
Professionals^c^	24 (7.8)
Administrative staff	26 (8.5)
Businessman	30 (9.8)
Others^d^	71 (23.3)
Education level	
High school and below	147 (48.1)
Junior college	83 (27.1)
Bachelor degree or above	76 (24.8)
Type of AL	
ALL	112 (36.6)
AML	194 (63.4)
Other disease^e^	
No	169 (55.2)
Yes	137 (44.8)
Disease course (month)	
≤ 6	181 (59.2)
> 6	125 (40.8)
Chemotherapy course (times)	
1 ~ 2	135 (44.1)
≥ 3	171 (55.9)
Treatment stage	
Induction	141 (46.1)
Consolidation	114 (37.2)
Maintenance^f^	51 (16.7)
Initial treatment or not	
Yes	235 (76.8)
No	71 (23.2)

*AL* Acute leukemia, *AML* Acute myelogenous leukemia, *ALL* Acute lymphoblastic leukemia

^a^ Not married includes single, widow, and divorced

^b^ Refer to practice a religion

^c^ Refer to engineers, doctors, nurses, teachers and other senior professional/technical workers

^d^ Others includes retirement

^e^ Refer to other primary diseases or chronic diseases except acute leukemia complications

^f^ Refer to support and symptomatic treatment of acute leukemia therapy

### Unmet supportive care needs of adult AL patients

Table 2 shows the unmet supportive care needs of adult AL patients. The 306 adult AL patients under study showed a variety of unmet SCNs, particularly in the health system and information, and psychological domains. The unmet demand rate for each of the 34-item unmet SCNs, ranges from 17.6 to 81.7%. The median (M) scores and IQR (P75–P25) of each domain are 53.41 (40.91) for the health system and information, 42.50 (37.50) for psychological, 40.00 (35.00) for physical/daily living, 35.00 (35.00) for patient care and support, and 0.00 (25.00) for the sexuality. A total of 42.8% of the subjects were categorized with “moderate or high demand” (unmet supportive care needs) as defined in health information domain followed by the psychological domain (29.5%), physical/daily living need domain (28.4%), care and support need domain (24.4%) and Sexual needs domain (5.7%) being the least (Table 3). Table 4 shows that, of the 10 highest unmet needs, six items are from the health system and information domain, and the top three items include: “being informed about things you can do to help yourself to get well” (81.7%), “being given information (written, diagrams, drawings) about aspects of managing your illness and side-effects at home” (77.5%), and “being adequately informed about the benefits and side-effects of treatments before you choose to have them” (70.3%). All three items with the lowest unmet requirements are from the sexual domain.

**Table 2:** Unmet supportive care needs and quality of life scores in all domains(n = 306)

Domain	Entry	Min	Max	Mean (SD)	M (IQR)
**FACT-Leu**					
Physical Well-Being	7	0.00	28.00	16.35(6.34)	17.00(9.00)
Social/Family Well-Being	7	5.00	28.00	18.28(5.02)	19.00(6.00)
Emotional Well-Being	6	0.00	24.00	16.19(4.85)	17.00(7.00)
Functional Well-Being	7	0.00	28.00	12.53(6.68)	13.00(10.00)
LEUS domain	17	4.00	65.00	40.62 (11.70)	42.00 (15.00)
Overall QOL	44	16.00	167.00	103.97 (26.27)	106.00 (32.25)
**Unmet SCNs**					
Health systems and information needs	11	0.00	100.00	54.78 (24.60)	53.41 (40.91)
Psychological	10	0.00	100.00	43.07 (24.24)	42.50 (37.50)
Physical/daily living	5	0.00	100.00	41.63 (23.35)	40.00 (35.00)
Patient care and support	5	0.00	95.00	38.35 (22.33)	35.00 (35.00)
Sexuality	3	0.00	100.00	14.73 (20.420	0.00 (25.00)

*FACT-LEU* Chinese Version of Functional Assessment of Cancer Therapy-Leukemia; The leukemia-specific subscale is called the LEUS domain in this article; Unmet SCNs: The Supportive Care Needs Survey form; *QOL* Quality of life, *SD* Standard deviation, *M* Median, *IQR* Interquartile range (P75-P25)

**Table 3:** Scoring level of unmet supportive care needs in each dimension (n = 306)

Domain of SCNs	Not Applicable (%)	Satisfield (%)	Low (%)	Moderate (%)	High (%)	Total^a^ (%)
Health systems and information	13.2	21.2	22.8	19.1	23.7	42.8
Psychological	26.4	16.7	27.3	17.8	11.7	29.5
Physical/daily living	28.3	16.2	27.1	17.3	11.1	28.4
Patient care and support	24.3	33.4	17.9	14.0	10.4	24.4
Sexuality	67.5	13.8	13.0	3.5	2.2	5.7

*SCNs* Supportive care needs

^a^ Sum of the percentage of moderate to high need

**Table 4:** Sequence of items of unmet supportive needs(*n* = 34)

Order^a^	Item	Domain	Mean (SD)	%^b^
1	Being informed about things you can do to help yourself to get well	I	3.81 (1.26)	81.70
2	Being given information (written, diagrams, drawings) about aspects of managing your illness and side-effects at home	I	3.22 (1.20)	77.50
5	Being adequately informed about the benefifits and side-effects of treatments before you choose to have them	I	3.38 (1.33)	70.30
6	Having access to professional counselling (e.g., psychologist, social worker, counselor, nurse specialist) if you, family or friends need it	I	3.13 (1.39)	69.60
9	Being informed about your test results as soon as feasible	I	3.40 (1.34)	68.30
10	Having one member of hospital staff with whom you can talk to about all aspects of your condition, treatment and follow-up	I	3.07 (1.34)	67.00
12	Being given written information about the important aspects of your care	I	2.96 (1.26)	65.00
13	Being informed about cancer which is under control or diminishing (that is, remission)	I	3.36 (1.37)	63.70
14	Being given explanations of those tests for which you would like explanations	I	3.22 (1.31)	63.10
20	Being treated in a hospital or clinic that is as physically pleasant as possible	I	2.94 (1.45)	52.90
27	Being treated like a person not just another case	I	2.61 (1.35)	43.50
3	Concerns about the worries of those close to you	P	3.39 (1.22)	76.80
4	Uncertainty about the future	P	2.87 (1.42)	72.20
8	Feelings about death and dying	P	2.54 (1.39)	68.60
11	Worry that the results of treatment are beyond your control	P	3.00 (1.34)	66.30
17	Feeling down or depressed	P	2.55 (1.23)	56.20
18	Learning of feel in control of your situation	P	2.71 (1.28)	54.90
19	Fears about the cancer spreading	P	2.62 (1.41)	53.90
21	Anxiety	P	2.45 (1.27)	52.00
22	Feelings of sadness	P	2.42 (1.22)	50.30
23	Keeping a positive outlook	P	2.61 (1.31)	49.70
7	Lack of energy/tiredness	H	3.00 (1.17)	68.60
15	Feeling unwell a lot of the time	H	2.76 (1.26)	61.80
16	Not being able to do the things you used to do	H	2.77 (1.39)	58.80
26	Pain	H	2.55 (1.42)	46.10
30	Work around the home	H	2.25 (1.34)	42.20
24	More choice about which cancer specialists you see	S	2.71 (1.44)	48.70
25	Hospital staff acknowledging, and showing sensitively to, your feelings and emotional needs	S	2.56 (1.75)	47.70
28	More choice about which hospital you attend	S	2.55 (1.39)	43.50
29	Hospital staff attending promptly to your physical needs	S	2.61 (1.24)	42.50
31	Reassurance by medical staff that the way you feel is normal	S	2.20 (1.08)	29.10
32	Being given information about sexual relationships	X	1.60 (1.03)	19.60
33	Changes in sexual feelings	X	1.59 (0.94)	18.60
34	Changes in your sexual relationships	X	1.57 (0.99)	17.60

*I* Health systems and information needs, *P* Psychological, *H* Physical/daily living, *S* Patient care and support, *X* Sexuality. *SD* Standard deviation

^a^This order refers to the order of each item in the original scale (SCNS-SF34).

^b^The unmet demand rate of each item = Number of patients with unmet support needs for this item/ Total number of patients who answered the item ×100%.

### Factors associated with unmet supportive care needs

Supplement 1 shows the results of the univariate analysis of this study, of which nine factors in the domains of unmet SCNs, including marital status, original residence, age, education, occupation, other diseases, chemotherapy course, disease course, and treatment stage. The assignment of each variable is shown in Supplement 2. Table 5 shows the factors associated with unmet supportive care needs of adult AL patients. Original residence, education level, occupation, other diseases, chemotherapy course, disease course and treatment stage were statistically significant in the unmet health system and information domain (*p* < 0.05). Marital status, education level, disease course and treatment stage have statistically significant differences in the psychological domain (*p* < 0.05). Occupation and treatment stage are significant in the physical/daily living domain (*p* < 0.05). Marital status, age, occupation, chemotherapy course, disease course and treatment stage are significantly associated with the level of patient care and support domain (*p* < 0.05). Age is significant in relation to patients’ sexual domains (*p* < 0.05).

**Table 5:** Multivariate logistic regression analysis on the factors of unmet supportive needs in all domains(*n* = 306)

Domain of SCNs	Variable	B	SE	Wald	*OR*	*95% CI*	*P*
Health systems and information	Occupation						
	Farmer	Ref	-	-	1.00	-	-
	Businessman	-1.71	0.65	6.95	0.18	0.05~0.65	0.008
	Other disease						
	No	Ref	-	-	1.00	-	-
	Yes	0.84	0.35	5.71	2.32	1.16~4.62	0.017
	Disease course (month)						
	≤6	Ref	-	-	1.00	-	-
	>6	-1.01	0.40	6.29	0.36	0.17~0.80	0.012
Psychological	Marital status						
	Married	Ref	-	-	1.00	-	-
	Not married	0.81	0.34	5.71	2.24	1.16~4.35	0.017
	Education						
	Bachelor degree or above	Ref	-	-	1.00	-	-
	Junior college	0.89	0.37	5.84	2.43	1.18~5.00	0.016
	High school and below	0.84	0.32	6.83	2.32	1.24~4.38	0.009
	Disease course (month)						
	≤6	Ref	-	-	1.00	-	-
	>6	-0.73	0.29	6.60	0.48	0.28~0.84	0.01
	Treatment stage						
	Induction	Ref	-	-	1.00	-	-
	Consolidation	-1.32	0.46	8.17	0.27	0.11~0.66	0.004
Physical/daily living	Occupation						
	Farmer	Ref	-	-	1.00	-	-
	Student	-1.02	0.42	5.96	0.36	0.16~0.82	0.015
	Professionals	-1.08	0.51	4.46	0.34	0.13~0.93	0.035
	Others	-0.77	0.35	4.86	0.46	0.23~0.92	0.027
	Treatment stage						
	Induction	Ref	-	-	1.00	-	-
	Consolidation	-0.79	0.28	7.87	0.45	0.26~0.79	0.005
Patient care and support	Marital status						
	Married	Ref	-	-	1.00	-	-
	Not married	0.93	0.31	8.92	2.53	1.38~4.65	0.003
	Age(year)						
	18~35	Ref	-	-	1.00	-	-
	>60	1.31	0.48	7.36	3.69	1.44~9.46	0.007
	Chemotherapy course (times)						
	1~2	Ref	-	-	1.00	-	-
	≥3	-1.05	0.34	9.51	0.35	0.18~0.68	0.002
	Treatment stage						
	Induction	Ref	-	-	1.00	-	-
	Consolidation	-0.73	0.26	8.04	0.48	0.29~0.80	0.010
Sexuality	Education						
	Bachelor degree or above	Ref	-	-	1.00	-	-
	Junior college	1.27	0.61	4.38	3.55	1.08~11.63	0.036

The results of first step regression analysis (including all variables) and the final model for each domain were given in ***supplement 1***

*SCNs* supportive care needs

### Association between unmet supportive care needs domains and quality of life

Table 2 shows that the mean score of overall QoL is 103 ± 26.27, with scores in each domain, ranked from high to low: SWB (18.28 ± 5.02), PWB (16.35 ± 6.34), EWB (16.19 ± 4.85), and FWB (12.53 ± 6.68) and LEUS domain (40.62 ± 26.27). The SCN domains (excluding social/family status) of adult AL patients negatively correlate with all QoL and overall QoL domains. Table 6 shows the correlations of unmet SCNs with QoL. Negative correlations are found in the overall QoL and physical/daily living, psychological, sexual, patient care and support, and health systems and information domains (r = − 0.527, *p* < 0.01; r = − 0.688, *p* < 0.01; r = − 0.170, *p* < 0.01; r = − 0.352, *p* < 0.01; r = − 0.220, *p* < 0.01).

**Table 6:** Spearman correlations (r) between unmet supportive needs and quality of life (n = 306)

Domain	PWB	SWB	EWB	FWB	LEUS domain	Overall QOL
Health systems and information needs	−0.259**	0.003	−0.183**	−0.135*	− 0.252**	−0.220**
Psychological	−0.594**	−0.066	− 0.702**	−0.469**	− 0.572**	−0.688**
Physical/daily living	−0.531**	−0.043	− 0.344**	−0.359**	− 0.505**	−0.527**
Patient care and support	−0.354**	−0.076	− 0.296**	−0.284**	− 0.301**	−0.352**
Sexuality	−0.271**	−0.064	− 0.168**	−0.132*	− 0.163**	−0.170**

## Discussion

To our knowledge, this is the first study in mainland China that has identified an association between unmet SCNs and the QoL of adult AL patients. The following three aspects will be discussed, based on the objectives and results of the research: (i) the unmet SCNs of adult AL patients, (ii) factors associated with the unmet SCNs of adult AL patients and (iii) the relationship between unmet SCNs and the QoL of adult AL patients in mainland China.

### The unmet SCNs of adult AL patients

The results show that the health systems and information domain scores the highest of the five domains, which is consistent with the results of previous studies [13, 37, 38]. Information needs are concerned with treatment for most cancer patients [39]. This may be because knowledge of disease-related information can affect patients’ confidence and self-efficacy in facing the disease, and also reflects their eagerness to understand initiatives to fight it. This serves as a reminder that healthcare professionals should pay more attention to providing adequate and effective informational support to patients, as they follow the disease trajectory.

The psychological domain of SCNs ranked second in this study, which is consistent with previous studies [13], with 76.8% patients reporting the item of “concerns about the worries of those close to you”. Previous studies [34, 40] have demonstrated that the item with the highest demand score in the psychological domain is “fear of cancer recurrence or spread”, which is inconsistent with the results of this study, and possibly due to the differences between AL and a solid tumour, or a limited sample size. The physical/daily living domain ranked third of the SCNs. Generally, ALs present several clinical symptoms, including fever, fatigue, bleeding, and infections, which is consistent with previous studies [41]. Long periods of hospitalization may also increase the psychological burden on patients.

In the patient care and support domain, our research finds the highest reported item is “more choice about which cancer specialists you see”, which may result from patients’ uncertainty regarding the treatment of the disease and their anticipated life expectancy. In terms of the sexual domain, the results of this study are consistent with previous research [13, 35]. For AL patients, treating the disease and managing symptoms is more important than their sexual lives, which can pose potential risks. The nature and the specificity of AL (e.g., bleeding, infection, etc.) may also increase patient concerns about the influence of the disease on their sexual life, in the context of Chinese culture.

The findings indicate that healthcare professionals should pay special attention to patients’ unmet supportive care needs, especially information and psychological needs. On the other hand, more effective interventions can be implemented to meet the needs of patients in a timely and effective manner.

### The factors associated with the unmet SCNs of adult AL patients

Our study reveals that the domains of the unmet SCNs of adult AL patients, depend on eight factors, including marital status, age, education level, occupation, presence of other diseases the disease course, chemotherapy course and treatment stage.

In terms of the participant’s marital status, SCNs are less for married people, which is consistent with the findings of previous studies [42]. However, it has been demonstrated that the emotional needs of divorced/widowed patients are significantly less than others, whereas the emotional needs of unmarried cancer patients remain unmet [35, 43]. These inconsistent results may be due to limited samples, or different cancer types. Previous literature on the impact of age on patient care, categorizes support needs differently. Our study finds that older patients (> 60 years old) demand more care and support than younger patients (18–35 years old). Some studies have shown that older people recover slowly during treatment because of their physical condition and, therefore, require additional care and support [44], whereas other studies report that younger patients have more unmet SCNs [45]. The probable cause may be greater demand from younger cancer patients for information about body image and interpersonal relationships.

In this study, education level was related to patients’ psychological and sexual needs. This may be because higher levels of education improve a patient’s self-efficacy and self-regulation ability. However, the evidence supporting the findings is limited. Our study finds that farmers have greater information needs than businessmen, similar to a previous study [46]. This may be partially explained by businessmen having greater access to social resources and additional channels to obtain information, than farmers. Further rigorous study is required to verify these findings.

Our research finds that the unmet needs of AL patients with other diseases, are greater than those of AL patients without other diseases, which is consistent with a previous study [47]. The results also indicate that the duration of the disease may affect unmet SCNs in consolidation and strengthening, which is consistent with a previous study [48]. Patients with fewer than two courses of chemotherapy show more unmet SCNs, which may be partially explained by the higher uncertainty of the disease experienced by patients during initial treatment, as it may raise concerns about the side effects. In this study, patients undergoing induced remission were at higher risk of unmet SCNs than those undergoing consolidation. Most patients with health system and information needs had relatively long disease courses (>6 months), which conflicts with a previous study [13]. Therefore, the evidence for a relationship between the disease course and adult AL patients, and unmet SCNs, requires further validation.

### The relationship between unmet SCNs and QoL in adult AL patients

Understanding the relationship between unmet supportive care needs and QoL, in adult AL patients, is a stepping stone to improving QoL [24]. This study shows that all domains of a patient’s unmet SCNs negatively correlate with QoL (excluding social/family status) and overall QoL. Although cancer patients’ QoL is influenced by the burden of disease-related symptoms and distress [37], unmet SCNs may partially explain the variance in QoL for adult AL patients. The more patients have unmet needs, the worse their QoL, as found by a previous study [49]. Additionally, the characteristics of susceptibility to recurrence, financial burden and high mortality, impair the mental health of patients and exacerbate their psychological needs. High levels of impaired physical and psychological unmet needs can severely affect a patient’s QoL [50]. One study suggests that clinicians can use needs assessment tools to assess patients’ QoL [51]. Early assessment of unmet supportive care needs and the provision of appropriate services can enhance patient QoL [52].

In summary, understanding the current unmet SCNs of patients can be a screening problem for patients, improving medical services and providing progress on the development of clinical care decisions and interventions [53]. Although the mechanisms of interaction between unmet SCNs and QoL are unclear, studies have shown that interventions for unmet SCNs in adult AL patients may be an effective method to improve their QoL [54]. The results of this study cannot be generalized to other types of cancer patients, who are entitled to a different cultural backgrounds and social security benefits.

### Study limitations

First, this study was conducted at three tertiary hospitals in Fuzhou, which may lead to institutional bias and limit the generality of the findings. Second, the study design is cross-sectional and may not be representative of the overall unmet SCNs of adult AL cancer patients in China. The cross-sectional design cannot establish causality. Third, patients’ unmet SCNs may be influenced by different cultural backgrounds and medical systems, limiting the generality of the result to populations of patients with other types of cancer. Finally, patients’ responses to unmet SCNs and QoL may be influenced by treatment regimens, side effects of chemotherapy, stressful life events, mental health at the time, and satisfaction with medical care.

### Implications for practice

The findings suggest some potential implications for practice. First, screening patients for unmet SCNs throughout treatment, is important for these patients to reduce their disease-related burden and risk of depression. Second, the factors affecting SCNs may be important entry points for nursing interventions. Healthcare professionals should detect patients’ unmet SCNs as early as possible, and provide targeted care for the influencing factors of unmet SCNs, to improve the QoL of adult AL patients. Finally, mobile medicine is strongly recommended to solve the problem of insufficient clinical nursing resources in meeting the unmet SCNs of patients.

## Conclusion

This study contributes to the knowledge of the relationship between unmet SCNs and the QoL of adult AL patients in China. Our results suggest that healthcare professionals need to seek effective interventions to meet the needs of patients, improve QoL, and intervene based on these underlying factors. Future research should explore other risk factors affecting SCNs at different stages of the disease, and to develop coordinated resources and programs to address the unmet SCNs of adult AL patients with different cultural characteristics.

## Supplementary information

**Additional file 1: Supplement 1.** Univariate analysis of supportive needs in patients with acute leukemia.

**Additional file 2: Supplement 2.** The included variable assignment of multiple regression

## Data Availability

The data that support the findings of this study are available on request from the corresponding author.

## Abbreviations

AL: Acute leukemia
ALL: Acute lymphoblastic leukemia
AML: Acute myelogenous leukemia
EWB: Emotional Well-Being
FACT-LEU: Chinese Version of Functional Assessment of Cancer Therapy-Leukemia
FWB: Functional Well-Being
IQR: Interquartile range (P75-P25)
LEUS domain: The leukemia-specific subscale of FACT-leu
PWB: Physical Well-Being
QoL: Quality of life
SCNs: Supportive care needs
SWB: Social/Family Well-Being
SD: Standard deviation
