# Supplementary material for: Unmet supportive care needs and its relation to quality of life among adult acute leukaemia patients in China: a cross-sectional study
Source: Health Qual Life Outcomes. 2020 Jun 23;18:199. doi: 10.1186/s12955-020-01454-5 (PMC7310469; doi:10.1186/s12955-020-01454-5)
Supplement: Supplementary file 1 — Additional file 1: Supplement 1. Univariate analysis of supportive needs in patients with acute leukemia. [file 12955_2020_1454_MOESM1_ESM.docx]

| **Supplement 1** Univariate analysis of supportive needs in patients with acute leukemia | | | | | | | | | | | | | | | | | | | | | |
| --- | --- | --- | --- | --- | --- | --- | --- | --- | --- | --- | --- | --- | --- | --- | --- | --- | --- | --- | --- | --- | --- |
| Variables | Health systems and information | | | | Psychological | | | | | Physical/daily living | | | | Patient care and support | | | | Sexuality | | | |
|  | No/low demand  n(%) | Moderate to high  n(%) | $\chi^{2}$ | *P* | No/low demand  n(%) | | Moderate to high n(%) | $\chi^{2}$ | *P* | No/low demand  n(%) | Moderate to high  n(%) | $\chi^{2}$ | *P* | No/low demand  n(%) | Moderate to high  n(%) | $\chi^{2}$ | *P* | No/low demand  n(%) | Moderate to high  n(%) | $\chi^{2}$ | *P* |
| ender |  |  |  |  |  | |  |  |  |  |  |  |  |  |  |  |  |  |  |  |  |
| Male | 36(54.5%) | 123(51.3%) | 0.23 | 0.635 | 49(57.0%) | | 110(50.0%) | 1.21 | 0.272 | 60(50.4%) | 99(49.6%) | 0.19 | 0.067 | 70(47.6%) | 89(56.0%) | 2.14 | 0.144 | 142(52.0%) | 17(51.5%) | 0.003 | 0.957 |
| Female | 30(45.5%) | 117(48.8%) |  |  | 37(43.0%) | | 110(50.0%) |  |  | 59(52.9%) | 88(47.1%) |  |  | 77(52.4%) | 70(44.0%) |  |  | 131(48.0%) | 16(48.5%) |  |  |
| Marital status |  |  |  |  |  | |  |  |  |  |  |  |  |  |  |  |  |  |  |  |  |
| Not married^a^ | 14(21.2%) | 65(27.1%) | 0.93 | 0.334 | 15(17.4%) | | 64(29.1%) | **4.38*** | ***0.036*** | 26(21.8%) | 53(28.3%) | 1.60 | 0.206 | 28(19%) | 51(32.1%) | **6.77**** | ***0.009*** | 69(25.3%) | 10(30.3%) | 0.39 | 0.533 |
| Married | 52(78.8%) | 175(72.9%) |  |  | 71(82.6%) | | 156(70.9%) |  |  | 93(78.2%) | 134(71.7%) |  |  | 119(81%) | 108(67.9%) |  |  | 204(74.7%) | 23(69.7%) |  |  |
| Original residence living place |  |  |  |  |  | |  |  |  |  |  |  |  |  |  |  |  |  |  |  |  |
| City | 45(68.2%) | 115(47.9%) | **8.52**** | ***0.004*** | | 51(59.3%) | 109(49.5%) | 2.36 | 0.125 | 69(58.0%) | 91(48.7%) | 2.53 | 0.112 | 83(56.5%) | 77(48.4%) | 2.00 | 0.160 | 145(53.1%) | 15(45.5%) | 0.69 | 0.405 |
| Country | 21(31.8%) | 125(52.1%) |  |  | 35(40.7%) | | 111(50.5%) |  |  | 50(42.0%) | 96(51.3%) |  |  | 64(43.5%) | 82(51.6%) |  |  | 128(46.9%) | 15(54.5%) |  |  |
| Religion |  |  |  |  |  | |  |  |  |  |  |  |  |  |  |  |  |  |  |  |  |
| No | 37(56.1%) | 121(50.4%) | 0.66 | 0.416 | 50(58.1%) | | 108(49.1%) |  |  | 60(50.4%) | 98(52.4%) | 0.12 | 0.735 | 76(51.7%) | 82(51.6%) | 0.00 | 0.982 | 139(50.9%) | 19(57.6%) | 0.52 | 0.47 |
| Yes | 29(43.9%) | 119(49.6%) |  |  | 36(41.9%) | | 112(50.9%) |  |  | 59(49.6%) | 89(47.6%) |  |  | 71(48.3%) | 77(48.4%) |  |  | 134(49.1%) | 14(42.4%) |  |  |
| Age (years) |  |  |  |  |  | |  |  |  |  |  |  |  |  |  |  |  |  |  |  |  |
| 14~35 | 36(54.5%) | 103(42.9%) | 2.83 | 0.243 | 46(53.5%) | | 93(42.3%) | 4.76 | 0.093 | 63(52.9%) | 76(40.6%) | 4.87 | 0.087 | 73(49.7%) | 66(41.5%) | **8.43*** | ***0.015*** | 119(43.6%) | 20(60.6%) | **6.54*** | ***0.038*** |
| 36~60 | 23(34.8%) | 106(44.2%) |  |  | 34(39.5%) | | 95(43.2%) |  |  | 45(37.8%) | 84(44.9%) |  |  | 64(43.5%) | 65(40.9%) |  |  | 116(42.5%) | 13(39.4%) |  |  |
| >60 | 7(10.6%) | 31(12.9%) |  |  | 6(7.0%) | | 32(14.5%) |  |  | 11(9.2%) | 27(14.4%) |  |  | 10(6.8%) | 28(17.6%) |  |  | 38(13.9%) | 0.00(0.0%) |  |  |
| Education |  |  |  |  |  | |  |  |  |  |  |  |  |  |  |  |  |  |  |  |  |
| High school and below | 25(37.9%) | 122(50.8%) | **6.37*** | ***0.041*** | 35(40.7%) | | 112(50.9%) | **8.05*** | ***0.018*** | 50(42.0%) | 97(51.9%) | 5.50 | 0.064 | 71(48.3%) | 76(47.8%) | 3.28 | 0.196 | 130(47.6%) | 17(51.5%) | 3.67 | 0.160 |
| Junior college | 17(25.8%) | 66(27.5%) |  |  | 20(23.3%) | | 63(28.6%) |  |  | 31(26.1%) | 52(27.8%) |  |  | 34(23.1%) | 49(30.8%) |  |  | 71(26.0%) | 12(36.4%) |  |  |
| Bachelor degree or above | 24(36.4%) | 52(21.7%) |  |  | 31(36.0%) | | 45(20.5%) |  |  | 38(31.9%) | 38(20.3%) |  |  | 42(28.6%) | 34(21.4%) |  |  | 72(26.4%) | 4(12.1%) |  |  |
| Occupation |  |  |  |  |  | |  |  |  |  |  |  |  |  |  |  |  |  |  |  |  |
| Farmer | 9(13.6%) | 72(30.5%) | **21.53**** | ***0.001*** | 17(19.8%) | | 64(29.6%) | 10.99 | 0.089 | 25(21.0%) | 56(30.6%) | **23.07**** | ***0.001*** | 41(27.9%) | 40(25.8%) | **14.21*** | ***0.042*** | 72(26.8%) | 9(27.3%) | 7.57 | 0.271 |
| General worker | 9(13.6%) | 26(11.0%) |  |  | 7(8.1%) | | 28(13.0%) |  |  | 4(3.4%) | 31(16.9%) |  |  | 10(6.8%) | 25(16.1%) |  |  | 28(10.4%) | 7(21.2%) |  |  |
| Student | 11(16.7%) | 28(11.9%) |  |  | 10(11.6%) | | 29(13.4%) |  |  | 22(18.5%) | 17(9.3%) |  |  | 23(15.6%) | 16(10.3%) |  |  | 36(13.4%) | 3(9.1%) |  |  |
| [Professionals](C:/Users/aaa/AppData/Local/youdao/dict/Application/8.5.3.0/resultui/html/index.html#/javascript:;)^b^ | 8(12.1%) | 14(5.9%) |  |  | 7(8.1%) | | 15(6.9%) |  |  | 12(10.1%) | 10(5.5%) |  |  | 9(6.1%) | 13(8.4%) |  |  | 22(8.2%) | 0(0.0%) |  |  |
| Administrative staff | 9(13.6%) | 20(8.5%) |  |  | 14(16.3%) | | 15(6.9%) |  |  | 11(9.2%) | 18(9.8%) |  |  | 16(10.9%) | 13(8.4%) |  |  | 24(8.9%) | 5(15.2%) |  |  |
| Businessman | 11(16.7%) | 14(5.9%) |  |  | 10(11.6%) | | 15(6.9%) |  |  | 12(10.1%) | 13(7.1%) |  |  | 19(12.9%) | 6(3.9%) |  |  | 23(8.6%) | 2(6.1%) |  |  |
| Others | 9(13.6%) | 62(26.3%) |  |  | 21(24.4%) | | 50(23.1%) |  |  | 33(27.7%) | 38(20.8%) |  |  | 29(19.7%) | 42(27.1%) |  |  | 64(23.8%) | 7(21.2%) |  |  |
| Other disease^c^ |  |  |  |  |  | |  |  |  |  |  |  |  |  |  |  |  |  |  |  |  |
| No | 46(69.7%) | 123(51.3%) | **7.12**** | ***0.008*** | 54(62.8%) | | 115(52.3%) | 2.77 | 0.096 | 73(61.3%) | 96(51.3%) | 2.95 | 0.086 | 86(58.5%) | 83(52.2%) | 1.23 | 0.268 | 153(56.0%) | 16(48.5%) | 0.68 | 0.409 |
| Yes | 20(30.3%) | 117(48.8%) |  |  | 32(37.2%) | | 105(47.7%) |  |  | 46(38.7%) | 91(48.7%) |  |  | 61(41.5%) | 76(47.8%) |  |  | 120(44.0%) | 17(51.5%) |  |  |
| Type of AL |  |  |  |  |  | |  |  |  |  |  |  |  |  |  |  |  |  |  |  |  |
| ALL | 26(39.4%) | 86(35.8%) | 0.28 | 0.595 | 32(37.2%) | | 80(36.4%) | 0.02 | 0.890 | 48(40.3%) | 64(34.2%) | 1.17 | 0.279 | 61(41.5%) | 51(32.1%) | 2.92 | 0.087 | 101(37.0%) | 11(33.3%) | 0.17 | 0.680 |
| AML | 40(60.6%) | 154(64.2%) |  |  | 54(62.8%) | | 140(63.6%) |  |  | 71(59.7%) | 123(65.8%) |  |  | 86(58.5%) | 108(67.9%) |  |  | 172(63.0%) | 22(66.7%) |  |  |
| Chemotherapy course (times) |  |  |  |  |  | |  |  |  |  |  |  |  |  |  |  |  |  |  |  |  |
| 1-2 | 21(31.8%) | 114(47.5%) | **5.16**** | ***0.023*** | 32(37.2%) | | 103(46.8%) | 2.32 | 0.128 | 51(42.9%) | 84(44.9%) | 0.13 | 0.723 | 49(33.3%) | 86(66.7%) | **13.35**** | ***0.000*** | 123(45.1%) | 12(36.4%) | 0.90 | 0.342 |
| ≥3 | 45(68.2%) | 126(52.5%) |  |  | 54(62.8%) | | 117(53.2%) |  |  | 68(57.1%) | 103(55.1%) |  |  | 98(54.1%) | 73(45.9%) |  |  | 150(54.9%) | 21(63.6%) |  |  |
| Initial treatment or not |  |  |  |  |  | |  |  |  |  |  |  |  |  |  |  |  |  |  |  |  |
| Yes | 47(71.2%) | 189(78.8%) | 1.67 | 0.197 | 60(69.8%) | | 176(80.0%) | 3.67 | 0.055 | 96(80.7%) | 140(74.9%) | 1.39 | 0.238 | 120(81.6%) | 116(73.0%) | 3.26 | 0.071 | 215(78.8%) | 21(63.6%) | 3.81 | 0.051 |
| No | 19(28.8%) | 51(21.3%) |  |  | 26(30.2%) | | 44(20.0%) |  |  | 23(19.3%) | 47(25.1%) |  |  | 27(18.4%) | 43(27.0%) |  |  | 58(21.2%) | 12(36.4%) |  |  |
| Disease course (month) |  |  |  |  |  | |  |  |  |  |  |  |  |  |  |  |  |  |  |  |  |
| ≤6 | 27(40.9%) | 154(64.2%) | **11.59**** | ***0.001*** | 43(50.0%) | | 138(62.7%) | **4.15*** | ***0.042*** | 71(59.7%) | 110(58.8%) | 0.021 | 0.884 | 78(53.1%) | 103(64.8%) | **4.34*** | ***0.037*** | 164(60.1%) | 17(51.5%) | 0.89 | 0.345 |
| >6 | 39(59.1%) | 86(35.8%) |  |  | 43(50.0%) | | 82(37.3%) |  |  | 48(40.3%) | 77(41.2%) |  |  | 69(46.9%) | 56(35.2%) |  |  | 109(39.9%) | 16(48.5%) |  |  |
| Treatment stage |  |  |  |  |  | |  |  |  |  |  |  |  |  |  |  |  |  |  |  |  |
| Induction | 22(33.3%) | 120(50.0%) | **7.77**** | ***0.021*** | 37(43.0%) | | 105(47.7%) | **7.41*** | ***0.025*** | 47(39.5%) | 95(50.8%) | **11.39**** | ***0.003*** | 58(39.5%) | 84(52.8%) | **8.53**** | ***0.014*** | 130(47.6%) | 12(36.4%) | 3.23 | 0.199 |
| Consolidation | 34(51.5%) | 80(33.3%) |  |  | 41(47.7%) | | 73(33.2%) |  |  | 58(48.7%) | 56(29.9%) |  |  | 67(45.6%) | 47(29.6%) |  |  | 97(35.5%) | 17(51.5%) |  |  |
| Maintenance^d^ | 10(15.2%) | 40(16.7%) |  |  | 8(9.3%) | | 42(19.1%) |  |  | 14(11.8%) | 36(19.3%) |  |  | 22(15.0%) | 28(17.6%) |  |  | 46(16.8%) | 4(12.1%) |  |  |
|  | | | | | | | | | | | | | |  |  |  |  |  |  |  |  |

**P＜0.05* ***P*＜0.01; AL: Acute leukaemia; AML: acute myelogenous leukaemia; ALL: acute lymphoblastic leukaemia

^a^ Not married includes single, widow, and divorced.

^b^ Refer to engineers, doctors, nurses, teachers and other senior professional/technical workers.

^c^ Refer to other primary diseases or chronic diseases except acute leukemia complications.

^d^ Refer to support and symptomatic treatment of acute leukemia therapy.
