# Supplementary material for: Unmet supportive care needs and its relation to quality of life among adult acute leukaemia patients in China: a cross-sectional study
Source: Health Qual Life Outcomes. 2020 Jun 23;18:199. doi: 10.1186/s12955-020-01454-5 (PMC7310469; doi:10.1186/s12955-020-01454-5)
Supplement: Supplementary file 2 — Additional file 2: Supplement 2. The included variable assignment of multiple regression [file 12955_2020_1454_MOESM2_ESM.docx]

**Supplement 2** The included variable assignment of multiple regression

| V[ariable](C:/Users/aaa/AppData/Local/youdao/dict/Application/8.5.3.0/resultui/html/index.html#/javascript:;) | Assignment |
| --- | --- |
| Marital status | 1= married，2=others |
| Age | 1= 18~35，2= 36~60，3= >60 |
| Original residence | 1=city，2=country |
| Education level | 1=bachelor degree or above，2=junior college，3= high school and below |
| Profession | 1=farmer，2=general worker，3=student，4=[professionals](C:/Users/aaa/AppData/Local/youdao/dict/Application/8.5.3.0/resultui/html/index.html#/javascript:;)，5=Administrative staff, 6=businessman，7=others |
| Other disease | 1=no，2=yes |
| Chemotherapy course | 1= 1~2，2= ≥3 |
| Disease course | 1= ≤6，2= >6 |
| Treatment stage | 1=Induction，2=Consolidation，3=Maintenance |
